# Supplementary material for: Causal interplay between lactose intolerance and gut microbiota: a combined bidirectional Mendelian randomization and in vivo validation study
Source: Front Nutr. 2026 Jun 1;13:1803337. doi: 10.3389/fnut.2026.1803337 (PMC13265576; doi:10.3389/fnut.2026.1803337)
Supplement: Supplementary file 1 [file Data_Sheet_1.zip › supplementary materials/Forward/forest plot/ebi-a-GCST90027779.finngen_R12_E4_LACTONAS.pdf]

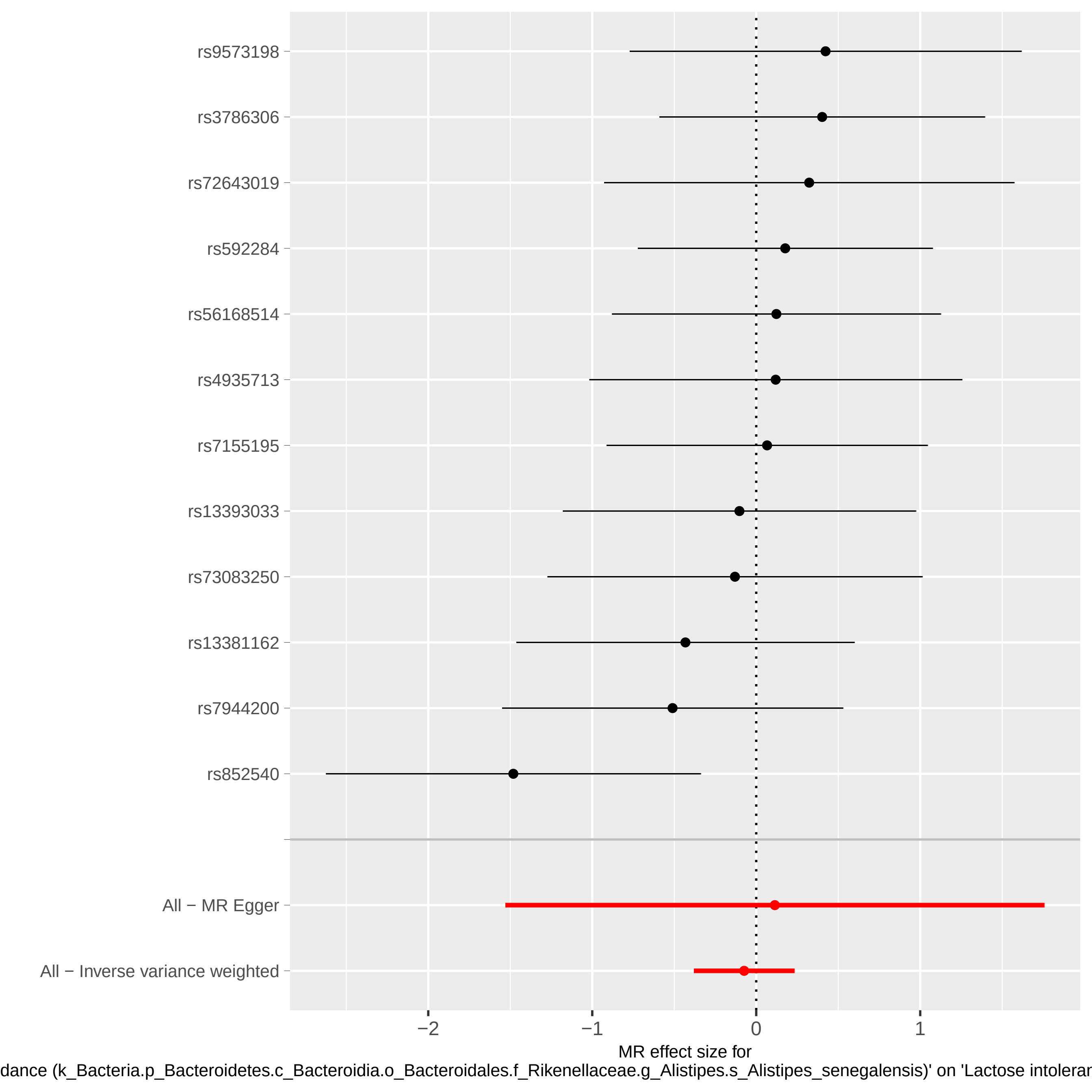
rs9573198
rs3786306
rs72643019
rs592284
rs56168514
rs4935713
rs7155195
rs13393033
rs73083250
rs13381162
rs7944200
rs852540
All − MR Egger
All − Inverse variance weighted
−2
−1
0
1
MR effect size for
dance (k_Bacteria.p_Bacteroidetes.c_Bacteroidia.o_Bacteroidales.f_Rikenellaceae.g_Alistipes.s_Alistipes_senegalensis)' on 'Lactose intoleran
